# A Tailored Physiotherapy Program in a Rare Case of Bilateral Territorial Cerebral Artery Infarction With Systemic Lupus Nephritis: A Case Report

**DOI:** 10.7759/cureus.54072

**Published:** 2024-02-12

**Authors:** Reva D Rajurkar, Nikita H Seth, Nishigandha P Deodhe, Neha Arya

**Affiliations:** 1 Neurophysiotherapy, Ravi Nair Physiotherapy College, Datta Meghe Institute of Higher Education and Research, Wardha, IND

**Keywords:** case report, physiotherapy, stroke, multimodal stimulation, physical therapy rehabilitation, systemic lupus erythema, bilateral cerebral artery infract

## Abstract

A stroke is a medical emergency that requires rapid treatment. Early intervention can help prevent brain damage and other negative consequences. An ischemic stroke occurs when a blood clot blocks or narrows a blood artery leading to the brain. Systemic lupus erythematosus (SLE) is characterized by systemic inflammation, autoantibodies, and a relapsing-remitting illness. Patients with SLE have an increased cumulative risk of cardiovascular events, including stroke, especially in the first five years after diagnosis. This case study presents the comprehensive physical therapy treatment of a 52-year-old woman diagnosed with systemic lupus nephritis and bilateral area infarct, an unusual combination. This condition resulted in issues such as decreased mobility, muscle weakness, and cognitive impairments. The specialized physiotherapy program employed a diverse strategy to address neurological weaknesses with multimodal stimulation, range of motion exercises, strength training, balance training, and more. The approach also includes energy-saving techniques and adaptive ways to manage symptoms associated with lupus nephritis to address fatigue and joint stiffness. The positive outcomes highlight the importance of tailored physical therapy regimens in maximizing functional recovery and improving the quality of life in the challenging context of concurrent bilateral infarct and systemic lupus nephritis. This case study emphasizes the need for individualized rehabilitation strategies in enhancing overall patient outcomes, contributing valuable insights to the sparse literature on managing such complex cases.

## Introduction

A stroke is caused when blood flow to the brain is disrupted, or there is abrupt bleeding in the brain. There are two types of strokes. An ischemic stroke occurs when blood flow to the brain is blocked. A hemorrhagic stroke refers to a stroke caused by abrupt bleeding in the brain [1]. Strokes were the primary cause of impairments and cognitive limitations, in addition to being the sixth most prevalent cause of death. Furthermore, 5.2% of global deaths were attributable to ischemic stroke [2]. Ischemic strokes are the most widespread type of stroke. Arteries may naturally narrow as people grow older, but some factors might potentially accelerate this process. These include smoking, hypertension, and obesity. Risk factors include high cholesterol, diabetes, and excessive alcohol consumption. Ischemic stroke can also be caused by atrial fibrillation, a type of irregular heartbeat. This can result in blood clots in the heart that break apart and enter the blood arteries that supply the brain [3]. Ischemic stroke and cerebral infarcts result in different degrees and types of brain injury, such as structural damage and lesions to the cerebral tissue, as well as impairments and death of neurons [2]. 

The Middle Cerebral Artery (MCA) territory is more frequently impacted by large-vessel ischemic strokes, whereas the Anterior Cerebral Artery (ACA) territory is less frequently affected. These blood vessels supply the internal capsule and caudate nucleus, as well as parts of the frontal, temporal, and parietal lobes [4]. The ACA stroke zone includes the superior and medial parietal lobes, as well as the midline of the frontal lobe. According to data from a series, these are uncommon causes of ischemic infarctions, accounting for 0.3% to 4.4% of all stroke cases [5]. Posterior cerebral artery (PCA) strokes can be difficult to diagnose due to the diversity of symptoms, which might be generic and inconsistent at first. This is further complicated by the fact that patients are not often aware of their symptoms, making it difficult to establish a timeframe. PCA strokes can affect multiple brain regions, including the occipital lobe, the inferomedial temporal lobe, a considerable portion of the thalamus, and the upper brainstem and midbrain [6].

Systemic lupus erythematosus (SLE) is characterized by systemic inflammation, autoantibodies, and a relapsing-remitting disease. SLE primarily affects young women and is distinguished by multisystem involvement. The central nervous system (CNS) is frequently targeted by SLE, which is associated with higher rates of morbidity and mortality [7]. Some of the most commonly affected organs are the skin, joints, heart, kidneys, and hematologic system [8,9]. Stroke is a common occurrence in SLE patients, affecting 3-20% of them. Most strokes occur within the first 5 years of diagnosis, particularly in younger people. SLE is usually diagnosed before a stroke occurs. SLE patients are 8 times more likely to have a stroke than the general population, and the risk of cardiovascular events increases by 3% each year following diagnosis. These factors contribute to 20-30% of SLE-related deaths [10]. SLE can cause cerebrovascular problems through various pathways, including hypercoagulable states, cardiogenic embolism, premature or accelerated atherosclerosis, and, in rare cases, vasculitis. SLE's clinical manifestation varies, as it can induce transient cerebral ischemia (TIA), arterial ischemic stroke, intraparenchymal hemorrhage, subarachnoid hemorrhage, and cerebral venous thrombosis [11].

An ischemic stroke can affect any of the territories supplied by the cerebral artery. In this case, the patient, known to have SLE, experienced damage to the anterior, posterior, and middle cerebral arteries bilaterally, resulting in the involvement of both the upper and lower limbs. Therefore, the goals of our rehabilitation were to improve the level of consciousness, prevent secondary complications, and enhance the function of both extremities. Painful sensory stimulation activates neurons in a vast network of brain locations involved in sensory and cognitive-affective processing, as well as nociception. Physiotherapy enhanced activity responsiveness. Physiotherapy may be appropriate to restore motor function and even cognition in patients with impaired consciousness [12].

## Case presentation

Patient information

A 52-year-old female was brought to the hospital by relatives with complaints of altered behavior, blurred vision for the past three days, and unresponsiveness for the last day. Upon arrival at the hospital, an MRI was performed, revealing an ischemic stroke. The patient was subsequently admitted to the ICU and was intubated. Antiplatelet medications, including clopidogrel (75 mg) and rosuvastatin, were administered. The patient has also had a known case of SLE for the past four years (no past investigations were available). She also had hypertension for six years and was on regular medication (Telmisartan 40 mg). Physiotherapy was recommended for the patient. After seven days, the patient was successfully extubated, and on the 15th day, she was transferred to the ward for further management. Rehabilitation was continued further.

Clinical findings

The patient was observed in a supine position. The level of consciousness was stupor, with a Glasgow Coma Score (GCS) of 8/15 and hemodynamically stable. On motor examination, the tone of both the upper and lower limbs was 3+ (hypertonia) on the Tone Grading Scale (TGS), which indicates an increase in muscle tone. The deep tendon reflexes for the biceps, triceps, supinator, knee, and ankle jerk were grade 3+ (exaggerated) on both sides. The Babinski sign was positive. Corneal and Doll's eye reflexes were also positive. Spasticity assessment was conducted according to the Modified Ashworth Scale (MAS) for bilateral upper and lower limbs; it was grade 1+ (slight increase in muscle tone, manifested as a catch, followed by minimal resistance through the remainder of the range of motion) [13].

Clinical diagnosis

**Figure 1:**
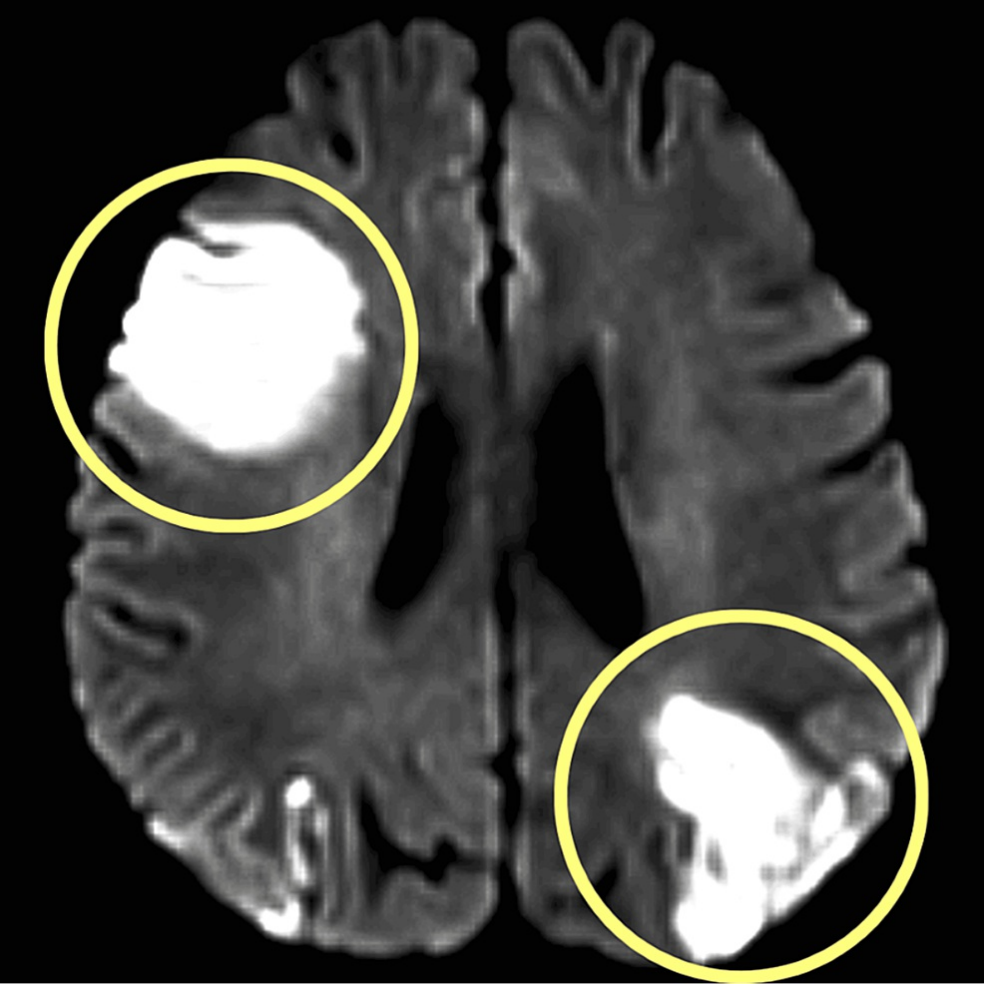
MRI findings. Yellow circles highlight acute infarct in the right frontal lobe, right insular cortex, bilateral parietal lobes, right posterior temporal lobe, and bilateral occipital lobes.

The patient was investigated with a routine checkup, MRI, and complete blood count (CBC). The MRI study depicts the following: acute infarct in the right frontal lobe, right insular cortex, bilateral parietal lobes, right posterior temporal lobe, and bilateral occipital lobes (Figure 1). Fluid-attenuated inversion recovery (FLAIR) hyperintensity in the left cerebellar hemisphere and gliotic changes. Chronic small vessel ischemic changes were seen. After all the investigations and clinical findings, the patient was diagnosed with MCA with ACA with PCA bilateral infarct with SLE.

Physiotherapy interventions

Physiotherapy rehabilitation primarily aimed at patient consciousness and secondary complications was planned for weeks 1 and 2, as detailed in Table 1. Caregivers were instructed to ensure proper positioning and constant changes in positioning every two hours. Physiotherapy rehabilitation targeting muscle weakness, hypertonia, bed mobility, and balance and coordination was planned for another four weeks, as outlined in Table 2.

**Table 1:** Physiotherapy treatment plan for weeks 1 and 2. DVT: Deep vein thrombosis.

Problem Identified	Goal	Treatment Strategy	Intervention
Patient and Caregiver Education	To encourage and maintain a positive perspective on therapy among the patient's family to promote early recovery.	Interaction between the patient, their family, and the therapist.	The patient's caregiver was well-informed about her condition and the importance of physiotherapy intervention. Explained all warning signs, dos, and don'ts.
Impaired Consciousness	To improve the level of consciousness, enhance sensory awareness, and assist the patient in being more aware of their own body.	Sensory stimulation, Proprioceptive and Tactile Stimulation (e.g., gentle tapping, auditory stimulation).	Various forms of sensory input (visual, auditory, and olfactory) can be used to stimulate the patient, such as applying ice, using textured materials, playing music, or providing familiar scents. The Rood's Approach (tapping on the belly and tendon), using pressure on the patient's limbs, or techniques such as massaging the skin to stimulate receptors beneath the skin.
Pressure Ulcer and Bedridden	To prevent pressure ulcers and other complications associated with prolonged immobility, such as DVT, contractures, etc.	Positioning and Bed mobility.	Regularly reposition the patient to avoid pressure ulcers and promote comfort, using pillows and changing positions every 2 hours. Bed mobility exercises, i.e., bed rolling from supine to side lying with assistance.

**Table 2:** Physiotherapy protocol for weeks 3 and 4.

Problem Identified	Goal	Treatment Strategy	Intervention
Hypertonia	To inhibit muscular tone and initiate movement in a pattern.	Rood's Inhibitory techniques, proprioceptive neuromuscular facilitation (PNF) patterns	Tone inhibitory techniques such as deep tendon pressure on the muscle tendon, gentle shaking on the muscle belly, joint compression held for 5-10 sec, and prolonged stretch for 30 sec for the upper and lower limb. (1 set of 10 repetitions), PNF technique of rhythmic initiation for upper and lower limbs (D1 and D2 patterns) (1 set of ten repetitions). Initially, it starts with passive range of motion (ROM), then progresses to active-assisted ROM, and then to active ROM.
Unable to Perform Bed Mobility Independently	To promote bed mobility	Functional mobility activities, bed mobility exercises	Supine lying to side lying, side lying to sitting at the edge of the bed, and gradually progress to standing beside the bed.
Impaired Balance and Coordination	To improve static and dynamic balance	Balance exercises (i.e., weight shifting, reach out, perturbation training)	For static balance, weight shifts side to side in sitting, reaches out in all directions, sit to stand (it started with support, progressing to without support). For dynamic balance, trunk PNF: stabilizing reversals and rhythmic stabilization (in sitting), perturbation from all directions.
Cognitive Impairments	To enhance cognitive functions	Cognitive training exercises	Memory, concentration, and problem-solving activities, as well as memory aids and tactics.
Emotional Distress	To improve emotional well-being	Counseling and psychotherapy	Relaxation techniques, offer emotional support and encouragement to cope with the psychological impact of stroke.

**Figure 2:**
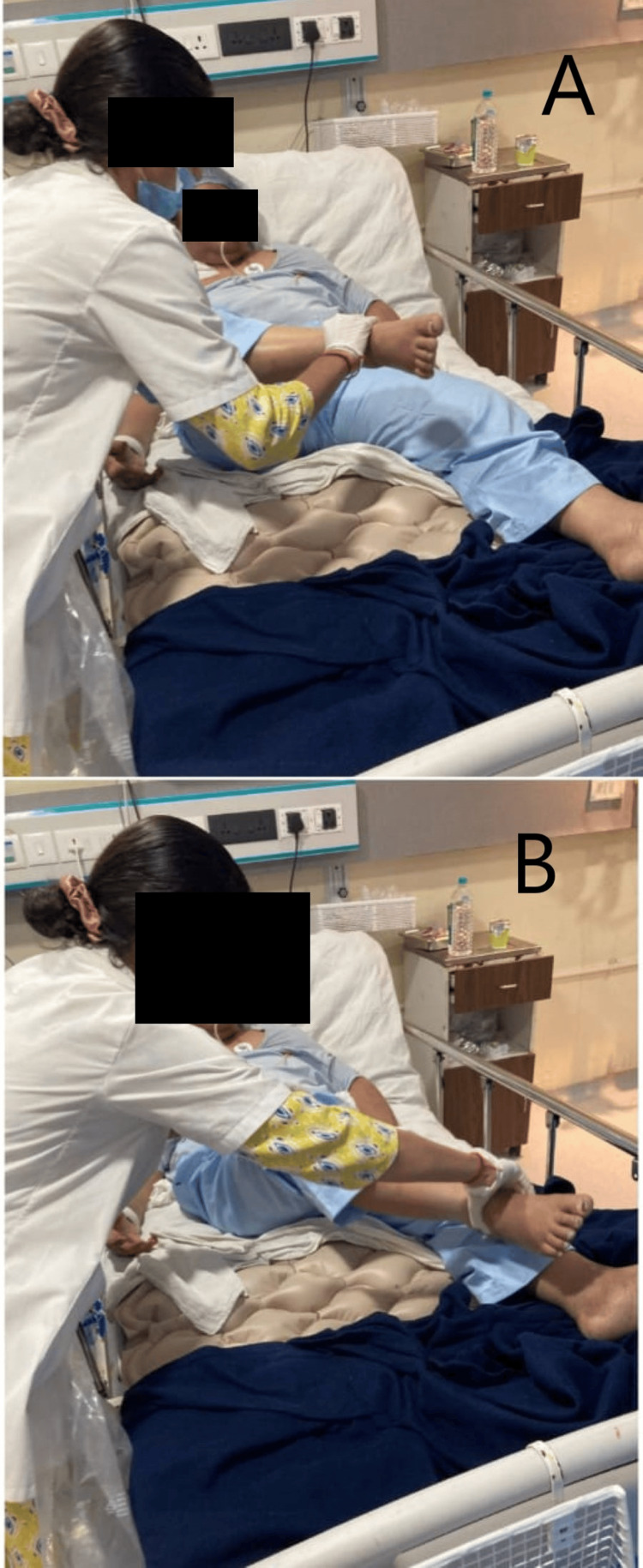
Patient being treated by PNF. A: Lower limb PNF D1 flexion pattern (rhythmic initiation) being performed. B: Lower limb PNF D1 extension pattern (rhythmic initiation) being performed. PNF: Proprioceptive neuromuscular facilitation; D1/2: Diagonal pattern.

The physiotherapy intervention received by the patient is summarized in Figure 2, which depicts the therapist providing a passive range of motion exercises using proprioceptive neuromuscular facilitation (PNF) D1 pattern for lower limb flexion to extension.

**Figure 3:**
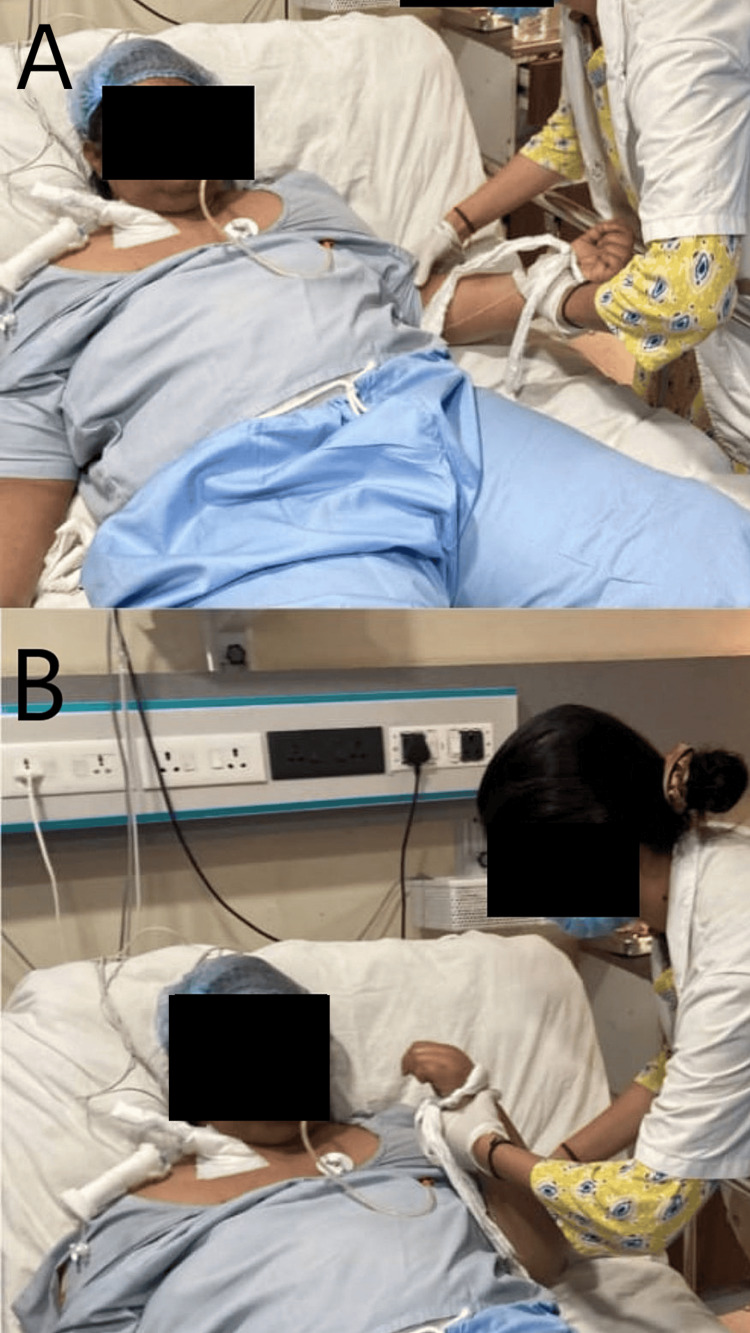
Elbow flexion-extension using the inhibitory approach A: Elbow extension achieved by applying deep tendon pressure on the triceps tendon. B: Elbow flexion achieved by applying deep tendon pressure on the biceps tendon.

Figure 3 illustrates the therapist applying the Rood inhibitory technique, i.e., deep tendon pressure to the biceps muscle for elbow flexion and extension.

Follow-up and outcome measures

An organized physical therapy intervention protocol was initiated. After four weeks of neuro-physiotherapy intervention, including multimodal stimulation, the patient regained consciousness. There was an improvement in spastic muscle tone, examined by the MAS for bilateral upper and lower limbs; it was assessed as grade 1 (slight increase in muscle tone, with a catch and release or minimal resistance at the end of the range of motion) [13]. The patient was able to sit with moderate assistance. Table 3 presents evaluation findings for outcome measures.

**Table 3:** Outcome measure pre and post-rehabilitation findings. GCS: Glasgow Coma Scale; FIM: Functional Independence Measure.

Sr no.	Outcome measures	Pre-score	Post-score
1.	GCS	8/15	13/15
2.	ICU mobility scale	0/10	3/10
3.	FIM	Level 2	Level 4
4.	Disability rating scale	2	3

## Discussion

Worldwide, acute ischemic stroke (AIS) is a leading cause of death and permanent disability, for which there are currently no effective treatments [14]. Compared to the general population or healthy controls, adult SLE patients have a two- to three-fold increased risk of stroke occurrences. The observed higher risk is likely related to known risk factors for stroke and variables associated with SLE [15]. Movement on both sides of the body is frequently hampered after a bilateral stroke. Improving the conditions of quadriplegic patients was the primary goal of this case study [16]. The case study showcases the comprehensive physical therapy treatment of a 52-year-old woman diagnosed with systemic lupus nephritis and bilateral area infarct, an unusual combination. This resulted in issues such as decreased mobility, muscle weakness, and cognitive impairments. The specialized physiotherapy program employed a diverse strategy to address neurological weaknesses with multimodal stimulation, range of motion exercises, strength training, balance training, and so on.

The application of motor learning concepts was evident in the structured rehabilitation program that targeted numerous physical and functional deficiencies in the presented case [16]. Leveraging the brain's innate neuroplasticity was a major component of the rehabilitation method, especially in terms of enhancing the patient's consciousness. The term neuroplasticity refers to the brain's ability to rearrange itself by forming new neural connections. Multimodal stimulation, which comprised auditory signals, visual stimuli, and mild tapping as sensory inputs, came into play at this point. These strategies aim to activate various sensory pathways, hence boosting neuron engagement and activation [16]. Furthermore, the intentional use of inhibitory methods addressed the observed increase in muscle tone, a common consequence of stroke. The technique of rhythmic initiation of PNF was utilized to reduce excessive muscle tone and elicit a more normalized muscle response [17]. This method supports adaptive changes in the brain circuits related to motor control, aligning with the principles of motor learning [18]. Previous research demonstrates enhanced function following physical therapy. The focus was on bilateral upper extremity and lower limb training, resulting in normalized tone, improved balance, etc. After an ischemic stroke, rigorous inpatient physical therapy promotes an early return to function [19].

## Conclusions

The rehabilitation plan that has been outlined concludes by highlighting the significance of a personalized and all-encompassing approach in the treatment of bilateral stroke. The goal of integrating various physiotherapeutic treatments with cognitive and emotional support is to optimize functional outcomes and enhance the overall quality of life for patients recovering from bilateral stroke. To ensure that interventions are customized to the patient's changing needs, regular reassessments and cooperation with a multidisciplinary team are crucial.
